# Cholera Epidemic in South Sudan and Uganda and Need for International Collaboration in Cholera Control

**DOI:** 10.3201/eid2405.171651

**Published:** 2018-05

**Authors:** Abdinasir Abubakar, Godfrey Bwire, Andrew S. Azman, Malika Bouhenia, Lul L. Deng, Joseph F. Wamala, John Rumunu, Atek Kagirita, Jean Rauzier, Lise Grout, Stephen Martin, Christopher Garimoi Orach, Francisco J. Luquero, Marie-Laure Quilici

**Affiliations:** World Health Organization, Cairo, Egypt (A. Abubakar);; Uganda Ministry of Health, Kampala, Uganda (G. Bwire, A. Kagirita);; Médecins Sans Frontières, Geneva, Switzerland (A.S. Azman);; Johns Hopkins Bloomberg School of Public Health, Baltimore, Maryland, USA (A.S. Azman, F.J. Luquero);; Epicentre, Paris, France (M. Bouhenia, L. Grout, F.J. Luquero);; Republic of South Sudan Ministry of Health, Juba, South Sudan (L.L. Deng, J. Rumunu);; World Health Organization, Juba (J.F. Wamala);; Institut Pasteur, Paris, France (J. Rauzier, M.-L. Quilici);; World Health Organization, Geneva (S. Martin);; Makerere University School of Public Health, Kampala (C.G. Orach)

**Keywords:** cholera, epidemic, Africa, South Sudan, Uganda, multilocus variable number tandem repeat, molecular microbiology, epidemiological data, bacteria, enteric infections, *Vibrio cholera*

## Abstract

Combining the official cholera line list data and outbreak investigation reports from the ministries of health in Uganda and South Sudan with molecular analysis of *Vibrio cholerae* strains revealed the interrelatedness of the epidemics in both countries in 2014. These results highlight the need for collaboration to control cross-border outbreaks.

Most countries in sub-Saharan Africa are affected by cholera epidemics ranging from annually to every 3–5 years or more (*1*,*2*). Cholera tends to be reported at the national or subnational level with few attempts to understand how it may simultaneously affect multiple countries in the same region (*3*–*5*). Published studies combining both microbiological and epidemiologic evidence are also scarce. However, better understanding of the interrelatedness of cholera spread between neighboring countries can provide the impetus for more cross-border collaboration in the fight against the disease. This subject is especially relevant in areas with porous borders that experience large population movements, like the border of South Sudan and Uganda.

Large urban communities or cities in cholera-prone areas may play a role in the persistence and transmission of cholera within Africa, given the high volume of travel between cities and other areas and the relatively high population density. Epidemiologic data do show support for the notion that large cities in Africa are hubs of transmission (*6*), but in-depth analyses are needed to substantiate or refute these hypotheses. Here we explore epidemiologic and microbiological data from cholera epidemics in Uganda and South Sudan in 2014 to establish possible interrelatedness.

## The Study

We used the official cholera dataset and outbreak investigation reports from the ministries of health in Uganda and South Sudan. Both countries use the Integrated Disease Surveillance and Response System (https://www.cdc.gov/globalhealth/healthprotection/idsr/about.html) and have adopted similar case definitions from the World Health Organization for areas with confirmed transmission. A suspected cholera case was defined as acute watery diarrhea in a person >2 years of age. A confirmed case was defined as a suspected case in which a stool sample had a culture-positive result for *Vibrio cholerae* O1 or O139.

**Figure 1:**
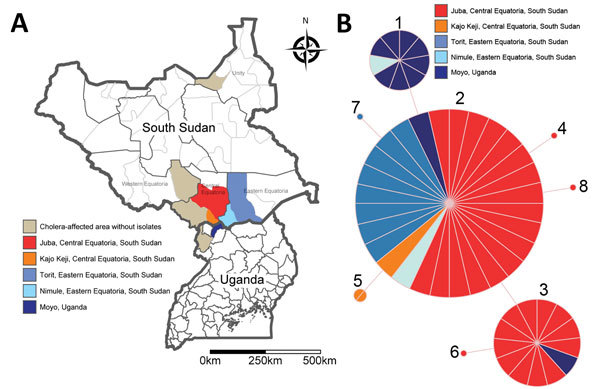
Locations and molecular analysis of 2014 epidemic in Uganda and South Sudan. A) Affected areas in both countries. Light brown indicates districts where we did not obtain any isolate for molecular analysis; red, orange, and blue areas represent affected districts with cholera isolates included in the analysis. B) Multilocus variable-number tandem-repeat (MLVA) analysis. Minimum spanning tree using pairwise difference was generated using Bionumerics version 6.6 (Applied Maths, Inc., Austin, TX, USA). Circles represent the 8 distinct MLVA profiles we identified, numbered chronologically by the earliest isolate of each profile. The MLVA profiles differ by variations at a single variable-number tandem-repeat locus located in the small chromosome only (VCA 0171, VCA 0283). The size of the circles is proportional to the number of isolates in each profile. Colors represent the location of the isolates and correspond with the colors of the areas in Panel A.

**Figure 2:**
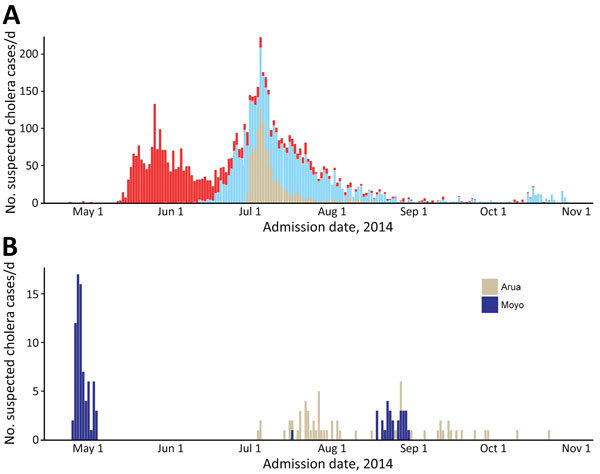
Epidemic curve of suspected cholera cases in South Sudan (A) and Uganda (B) in 2014, by hospital admission date and region.

Cholera case reporting began on April 25, 2014, in Moyo District in northern Uganda, bordering with Kajo-Keji County in South Sudan (Figure 1, panel A). This region reported 88 cases and 3 deaths in the subsequent weeks (Figure 2). The epidemic was contained after rapid implementation of control measures.

Cholera case reporting began on April 29, 2014, in Juba, South Sudan (300 km from Moyo town, Uganda), with a case investigation finding no evidence of travel outside of Juba. Within days, the first reported cholera outbreak in South Sudan since 2009 began, resulting in 6,269 suspected cases, including 105 deaths in health facilities and 51 community deaths (case-fatality ratio 2.4%) by the end of October 2014 (*7*). Transmission continued in Juba throughout the epidemic, and outbreaks occurred throughout the country, including large outbreaks in the north.

In early July 2014, months after the last confirmed case in Uganda but during a period of intense transmission in South Sudan, a new outbreak was reported in Moyo district, in subcounties (Metu and Dufile) that were not affected during the first outbreak (Itirikwa, Aliba, and Gimara); cases were eventually reported in the neighboring Arua district. An investigation revealed that the presumed index case-patient of this second outbreak had traveled to South Sudan. In total, 86 cases and 4 deaths were reported in Moyo and Arua Districts by October 22.

Although not all suspected cases were confirmed during the outbreaks, both countries routinely sent samples to their respective national reference laboratory for microbiological confirmation. We characterized 56 strains at Institut Pasteur (Paris, France) by determining the antimicrobial drug resistance patterns using the disk diffusion method following CA-SFM (Comité de l'Antibiogramme de la Société Française de Microbiologie) 2013 standards for *Enterobacteriaceae* (http://www.sfm-microbiologie.org/); subtyping with pulse-field gel electrophoresis (*8*) with *Sfi*I and *Not*I restriction enzymes (Roche Molecular Biochemicals, Indianapolis, IN, USA); and multilocus variable number tandem repeat (MLVA) analysis targeting 6 loci in the *V. cholerae* genome (*9*), and by genotyping tests (*10*)(Table). We used BioNumerics version 6.6 (Applied Maths, Inc., Austin, TX, USA) for clustering analysis (Technical Appendix).

**Table:** Characterization of *Vibrio cholerae* O1 isolates from South Sudan and Uganda, 2014*

Location	No. isolates*	Sample collection period	MLVA profile no.	VNTR loci designation						PFGE profile†	
				VC 0147	VC 0437	VC 1457	VC 1650	VCA 0171	VCA 0283		
Uganda and South Sudan	9	2014 April–June	1	10	7	3	7	10	18	1/1 (8), 2/1 (1)	
Uganda and South Sudan	28	2014 May–July	2	10	7	3	7	9	18	1/1	
Uganda and South Sudan	13	2014 May–July	3	10	7	3	7	9	19	1/1	
South Sudan	1	2014 May	4	10	7	3	7	9	20	1/1	
South Sudan	2	2014 June	5	10	7	3	7	9	17	1/1	
South Sudan	1	2014 June	6	10	7	3	7	10	19	1/1	
South Sudan	1	2014 June	7	10	7	3	7	11	18	1/1	
South Sudan	1	2014 July	8	10	7	3	7	9	16	1/1	

*MLVA, multilocus variable-number tandem-repeat analysis; PFGE, pulsed-field gel electrophoresis; VNTR, variable-number tandem-repeat. †PFGE profile number obtained after restriction with *Not*I and *Sfi*I respectively (*Not*I/*Sfi*I). The number in parentheses refers to the number of isolates sharing the same profile.

All 56 isolates were *V. cholerae* O1 serotype Inaba, atypical El Tor biotype, based on *rstR*^ET^, *tcpA*^ET^, and *ctxB*^Cla^ gene sequences (classical *ctxB1* allele) (*10*). The isolates shared similar antimicrobial drug resistance patterns and were resistant to trimethoprim/sulfamethoxazole, sulfonamides, streptomycin, and nalidixic acid (confirmed by MIC determination [MICs 16–256 mg/L] with Etest; AB bioMérieux, Solna, Sweden). Sequencing of the genes encoding DNA gyrase (*gyrA* and *gyrB*) and topoisomerase IV (*parC* and *parE*) detected 1 mutation in *gyrA* (substitution of serine by isoleucine at position 83), which has been associated with quinolone resistance in clinical *V. cholerae* isolates (*11*). PFGE analyses revealed a single *Sfi*I profile and 2 *Not*I profiles, 1 represented by a single strain. We identified 8 highly related MLVA profiles (Figure 1, panel B) and found variability only in 2 loci on the small chromosome (VCA0171 and VCA0283). All MLVA profiles formed a single clonal complex, in which all isolates can be connected through mutations at a single locus. These results demonstrate genetic uniformity of isolates and provide strong evidence that these epidemics in 2 countries resulted from the spread of a single clone with probable epidemiologic links.

This analysis has several limitations. The identified index cases in each country may not truly have been the initial case-patients, given that the surveillance systems in both locations are not highly sensitive. We selected the cases for culture by convenience sampling. Whereas random sampling is ideal, it is difficult to implement during epidemics because of competing priorities. Furthermore, although studies have shown that 6-locus MLVA can be highly discriminative for identifying a closely related pandemic strain isolated in a small timeframe and geographic area (*12*,*13*), our lack of knowledge of MLVA limitations makes it more difficult to draw inferences about strain relatedness and phylogenetic history, especially compared with whole-genome sequencing (*14*).

## Conclusions

Through epidemiologic and molecular data, we illustrated that the 2 outbreaks in South Sudan and Uganda in 2014 clustered into a single epidemic. The spread of cholera from border communities in Uganda to South Sudan and from South Sudan back to Uganda is a critical issue that needs further clarification to improve control strategies. Isolating the neighboring communities from one another is not possible; however, we recommend coordinated interventions by the 2 countries to identify the sources of infection, as was done during Ebola outbreaks in West Africa during 2014–2016.

The 2014 cholera epidemics probably evolved from a local outbreak in northern Uganda to a national outbreak in South Sudan; population movement, living conditions, and events in the capital, Juba, most likely played a key role in the spread of the disease to other areas in South Sudan and beyond. Refining our understanding of cholera beyond administrative boundaries, perhaps adopting regional approaches in addition to national cholera control efforts, and including key hubs of transmission, such as cities, may be key to minimizing the spatial extent and magnitude of future epidemics.

Joint implementation of disease control interventions and rapid information sharing platforms can strengthen collaboration between states to control the outbreaks. Further studies to describe the relatedness and routes of transmission of *V. cholerae* organisms and track the progression of the outbreaks, combining traditional and molecular epidemiologic tools, can aid public health decision making in Africa and beyond (*15*). International agencies should facilitate funding and support for joint country activities to expedite control of cross-border cholera epidemics.

Technical Appendix More information about the analyses performed in study of cholera outbreaks in South Sudan and Uganda in 2014.
