# Supplementary material for: Cholera Epidemic in South Sudan and Uganda and Need for International Collaboration in Cholera Control
Source: Emerg Infect Dis. 2018 May;24(5):883–7. doi: 10.3201/eid2405.171651 (PMC5938777; doi:10.3201/eid2405.171651)
Supplement: Technical Appendix — More information about the analyses performed in study of cholera outbreaks in South Sudan and Uganda in 2014. [file 17-1651-Techapp-s1.pdf]

Article DOI: https://doi.org/10.3201/eid2405.171651

Cholera Epidemic in South Sudan and Uganda and Need for International Collaboration in Cholera Control

Technical Appendix

Multilocus Variable Number Tandem Repeat Analysis for 6 Loci of *V. cholerae*

We selected 6 variable number tandem repeat (VNTR) loci, VC0147, VC0437, VC1457, VC1650, VCA0171 and VCA0283, previously identified and studied by Danin-Poleg et al. (*9*). We amplified the loci by PCR using their published primer sequences with some modifications: we performed 2 multiplexed PCR assays with fluorescently labeled primers. Mix 1 included VC0437, VC1650, and VCA0283, with final concentrations of primers of 0.8 µM, 0.6 µM, and 1.4 µM respectively and an annealing temperature of 60°C. Mix 2 included VC0147, VC1457, and VCA0171with final concentrations of 0.4 µM for each primer and an annealing temperature of 62°C. Fluorescent dyes were FAM for blue and PET for red fluorescence. We subjected PCR products to capillary electrophoresis on an ABI 3730XL DNA sequencer (Eurofins Genomics, Paris, France). We determined the sizes of PCR fragments with the GeneMapper v4.0 software (Applied Biosystems, Foster City, CA, USA). We assigned an allele number corresponding directly to the number of tandem repeats to each PCR fragment. We then imported allele strings into a BioNumerics database and constructed a minimum spanning tree (BioNumerics v. 6.6, Applied Maths, Inc., Austin, TX, USA).

Pulsed-Field Gel Electrophoresis

We typed each isolate according to PulseNet standardized procedures with the *Not*I and *Sfi*I restriction enzymes (*8*). We performed image acquisition and pattern analysis using

BioNumerics v. 6.6 software and visually, due to the similarity of the patterns. All the outbreak strains except one shared a unique pattern, the latter differing only by 2 bands after restriction with *NotI*. We assigned a number to each of the 2 different pulsotypes.
